# Trefoil factor 3 as a marker of gastrointestinal cell injury during sepsis

**DOI:** 10.1007/s00431-026-07060-9

**Published:** 2026-05-19

**Authors:** Ola Galal Ali Behairy, Amira Osama Abd El-Gaffar, Basma Galal Ali, Eman Sobhy Abdel-Gayed, Rasha Mohammed Zakaria

**Affiliations:** 1 Faculty of Medicine, Benha University, Benha, Egypt; 2 Clinical and Chemical Pathology, Faculty of Medicine, Benha University, Benha, Egypt

**Keywords:** Trefoil factor 3, TFF3, Gastrointestinal cell injury, Sepsis, Children

## Abstract

The purpose of this study is to evaluate trefoil factor 3 (TFF3) as a biomarker of gastrointestinal cellular injury in children with sepsis. This case–control study included 100 children admitted to the Pediatric Intensive Care Unit (PICU) with sepsis. They were divided into two groups according to gastrointestinal involvement, in addition to 30 age- and sex-matched healthy children who served as a control group. All participants underwent laboratory investigations, including complete blood count (CBC), liver, and kidney function tests. The Pediatric Logistic Organ Dysfunction (PELOD) score was applied to assess organ dysfunction severity, and TFF3 levels were measured using the enzyme-linked immunosorbent assay (ELISA) technique. TFF3 levels were markedly elevated in sepsis with gastrointestinal involvement compared to sepsis without gastrointestinal involvement and controls (*P* < 0.001). In these patients, levels were significantly higher in those with ileus, bleeding per rectum, and melena compared to those without. Non-survivors had higher TFF3 levels than survivors. Regarding blood culture results, patients with *Klebsiella* had the highest TFF3 levels compared to those with *Staphylococcus aureus* and those with no growth. In the multinomial logistic regression analysis using the control group as the reference category, TFF3 emerged as a significant predictor of sepsis outcomes. For patients with sepsis without gastrointestinal involvement, higher TFF3 levels were significantly associated with increased odds of sepsis (OR = 1.814, 95% CI 1.282–2.567, *P* = 0.001). Among patients with sepsis and gastrointestinal involvement, TFF3 demonstrated a more pronounced association (OR = 3.095, 95% CI 2.044–4.686, *P* < 0.001).

*Conclusion:* TFF3 is a valuable tool in the PICU setting, providing an objective measure of intestinal damage and assisting clinicians in the anticipation, diagnosis, and management of gastrointestinal failure in vulnerable pediatric patients.

**Table:** 

**What is Known:** • *Trefoil factor 3 (TFF3) is a mucosal protective peptide involved in epithelial restitution and gut barrier integrity and has been investigated as a potential biomarker of intestinal injury.*	
**What is New:** • *This study demonstrates that serum TFF3 levels are significantly elevated in pediatric sepsis, particularly in children with gastrointestinal involvement, ileus, rectal bleeding, or melena.* • *Elevated TFF3 levels were associated with poor outcomes and independently predicted sepsis severity and gastrointestinal dysfunction in critically ill children.*

**What is Known:**

• *Trefoil factor 3 (TFF3) is a mucosal protective peptide involved in epithelial restitution and gut barrier integrity and has been investigated as a potential biomarker of intestinal injury.*

**What is New:**

• *This study demonstrates that serum TFF3 levels are significantly elevated in pediatric sepsis, particularly in children with gastrointestinal involvement, ileus, rectal bleeding, or melena.*

• *Elevated TFF3 levels were associated with poor outcomes and independently predicted sepsis severity and gastrointestinal dysfunction in critically ill children.*

## Introduction

Sepsis is a severe, life-threatening clinical condition characterized by organ dysfunction caused by a dysregulated host immune response to infection. This abnormal systemic inflammatory reaction may advance to septic shock and ultimately lead to multiple organ dysfunction syndrome (MODS) [1].

Among the organ systems affected by sepsis-induced multiorgan dysfunction, the gastrointestinal tract is considered a major target. The integrity of the intestinal barrier, maintained by tight junctions between epithelial cells and an overlying protective mucus layer, ensures selective permeability while restricting the passage of luminal antigens, microorganisms, and their toxins into the systemic circulation [2].

Intestinal ischemia–reperfusion constitutes a frequent pathophysiological mechanism underlying various conditions in infants, children, and adults and may ultimately contribute to multiple organ dysfunction syndrome and increased mortality. The etiology of intestinal ischemia is multifactorial, ranging from generalized hypoperfusion to regional reductions in blood flow within the splanchnic circulation. In the context of sepsis, intestinal ischemia can occur due to impaired nutrient extraction and utilization by the intestine despite adequate oxygen content and delivery [3].

Trefoil factor 3 is a protein essential for the preservation and protection of mucosal surfaces throughout the body, particularly within the gastrointestinal tract. It is a member of the trefoil factor family, a group of three small cysteine-rich secreted peptides—TFF1, TFF2, and TFF3—synthesized by specialized mucus-secreting epithelial cells [4].

TFF3 is a peptide secreted by goblet cells of the gastrointestinal tract and is involved in mucosal defense, epithelial restitution, and the preservation of mucosa integrity [5].

Its expression can be altered in response to sepsis-induced gut barrier dysfunction, making it a potential diagnostic and prognostic tool. Further research into the precise mechanisms and therapeutic uses of TFF3 in sepsis-related intestinal damage is ongoing and holds promise for improving the management of this life-threatening condition [6].

This study aimed to evaluate trefoil factor 3 (TFF3) as a biomarker of gastrointestinal cellular involvement in pediatric sepsis.

## Patients and methods

This case control study was conducted at a Pediatric Intensive Care Unit. The study protocol was reviewed and approved by the local ethics committee of the Faculty of Medicine, Benha University from January 2024 to November 2024 (Approval No. MS 36—12—2023). This study was done in accordance with the Declaration of Helsinki.

Following a detailed explanation of the study objectives and procedures, a written informed consent was obtained from the legal guardians of all participants before enrollment.

### Inclusion criteria

The present study was carried out on 100 children, aged from 1 month to 16 years of both sex, admitted to PICU with sepsis, and stayed for at least 1 week; patients were categorized into two groups based on the presence or absence of gastrointestinal involvement, in addition to 30 healthy children matched for age and sex who served as a control group.

Gastrointestinal (GIT) injury in pediatric sepsis is currently best defined within the framework of pediatric organ dysfunction using the Pediatric Organ Dysfunction Information Update Mandate (PODIUM) criteria. According to this recent consensus, severe gastrointestinal dysfunction is characterized by objective evidence of intestinal injury, including bowel ischemia, perforation, pneumatosis intestinalis, or sloughing of intestinal mucosa confirmed by imaging or surgical findings, reflecting disruption of gut barrier integrity and contributing to systemic inflammation and multiple organ dysfunction. However, no standardized or validated criteria exist for mild or moderate gastrointestinal dysfunction in children, and commonly used clinical manifestations such as ileus, feeding intolerance, abdominal distension, or gastrointestinal bleeding remain non-specific and largely unvalidated for defining organ dysfunction in pediatric sepsis [7].

### Exclusion criteria

Children with pre-existing gastrointestinal conditions (e.g., inflammatory bowel disease) and chronic conditions (e.g., chronic kidney disease, liver cirrhosis).

### Study groups

Patients were divided into two groups according to gastrointestinal involvement; thus, we had three groups in this study:

Sepsis with gastrointestinal involvementgroup included 37 children (37%) (26 males and 11 females); their median age was 4 years.

Sepsis without gastrointestinal involvementgroup included 63 children (32 males and 31 females); their median age was 3 years.

- Control group included 30 children (17 males and 13 females); their median age was 3.5 years.

### Sample size

This study was designed in accordance with the methodology described by Žurek et al. [3]; sample size estimation was conducted using Epi Info STATCALC, assuming a two-sided confidence level of 95%, a study power of 80%, and a margin of error of 5%. Based on the software output, the calculated maximum required sample size was 70 participants.

### Methods

All enrolled patients underwent comprehensive history taking, thorough clinical examination, and laboratory investigations, including complete blood count (CBC) and assessment of liver and kidney function tests.

Seven milliliters of venous blood was obtained under aseptic conditions by clean venipuncture using a sterile disposable plastic syringe and was processed for the following investigations:

Complete blood count was assessed by utilizing the automated hematology analyzer, XS series, model SN 12526, manufactured by SYSMEX Corporation, Kobe, Japan.

Liver function tests (aspartate-aminotransferase (AST), alanine aminotransferase (ALT)) and kidney function test (urea and creatinine) were assessed by DIALAB, 13,771,103, Thermo Company, USA.

Blood samples for the measurement of trefoil factor 3 (TFF3) were obtained at the time of PICU admission (within the first 24 h of diagnosis of sepsis), prior to initiation of major therapeutic interventions. TFF3 levels were calculated using a Human TFF3 ELISA Kit (Catalogue No. 201—12—5153, SunRed Biotechnology Co., Shanghai, China) following the manufacturer’s instructions. In brief, standard, blank, and samples were added to the wells of the microplate. Standard wells received 50 µL of standard and 50 µL of streptavidin-HRP, while test wells received 40 µL of sample followed by 10 µL of TFF3 antibody and 50 µL of streptavidin-HRP. Blank wells contained only chromogen solutions and stop solution. After gentle shaking, the plate was incubated for 60 min at 37 °C. The supernatant was carefully discarded, and the plate was washed thoroughly. Subsequently, 50 µL of chromogen solution A and 50 µL of chromogen solution B were added to each well, followed by incubation for 10 min at 37 °C in the dark. The reaction was completed by adding 50 µL of stop solution to each well. Optical density (OD) was measured at 450 nm within 15 min by the blank well as zero. TFF3 concentrations were calculated from the standard curve generated by linear regression analysis and expressed as ng/mL.

The Pediatric Logistic Organ Dysfunction (PELOD) score was utilized to assess severity of organ dysfunction. The PELOD score is a widely applied clinical tool in Pediatric Intensive Care Units (PICUs) for evaluating the extent of multiple organ dysfunction and estimating the risk of mortality among critically ill children [8].

All GI complications were recorded and defined as follows [9]:

**Table:** 

**Vomiting**	Involuntary, forceful expulsion of gastric contents
Ileus	Inability to tolerate oral intake caused by impaired gastrointestinal motility without evidence of mechanical obstruction
Diarrhea	Passage of more than four stools per day or a noticeable change in stool consistency
Hematemesis	Vomiting of blood, which may appear bright red or resemble coffee grounds
Melena	Passage of black, tarry stools
Bleeding per rectum	presence of bright red blood exiting from the anus
Constipation	No stool for more than 48 h
Abdominal distension	Visible enlargement of the abdomen and/or an increase in abdominal girth of ≥ 2 cm from baseline, or radiographic evidence of significantly dilated bowel loops on abdominal X-ray

### Outcome

The primary outcome of this study was to evaluate the diagnostic value of TFF3 in identifying gastrointestinal involvement in pediatric sepsis and differentiating septic patients from healthy controls.

Secondary outcomes included assessing the association of TFF3 levels with clinical severity (PELOD score), gastrointestinal manifestations, length of hospital stay, and patient outcomes (survival vs non-survival), as well as evaluating its predictive performance using ROC curve and regression analyses.

### Statistical methods

Data management and statistical analysis were conducted using SPSS version 27 (IBM, Armonk, NY, USA). Quantitative variables were evaluated for normality using the Shapiro–Wilk test and visual inspection of the data. Based on the distribution, quantitative data were presented as mean ± standard deviation or median with range. Numbers and percentages were used to summarize categorical data. Quantitative data were compared among the three groups using one-way ANOVA test and Kruskal–Wallis test for parametric and non-parametric variables, respectively. Independent *T*-test and Mann–Whitney *U* test were employed to compare quantitative data between the two septic groups for parametric and non-parametric variables, respectively. Categorical data were compared using the chi-square or Fisher’s exact test. Correlations between trefoil factor 3 and other parameters in septic patients with and without gastrointestinal involvement were done using Spearman’s correlations. ROC analyses were performed for trefoil factor 3 to differentiate between (1) septic patients with gastrointestinal involvement and controls and (2) septic patients with and without gastrointestinal involvement. For each ROC analysis, the area under the curve with 95% confidence intervals, optimal cutoff points, and diagnostic indices were calculated. Multivariate linear regression analysis was conducted to predict the PELOD score. The regression coefficients along with their 95% confidence intervals were determined. All analyses were two-tailed, and *P*-values under 0.05 were regarded as significant.

## Results

The sepsis group with gastrointestinal involvement included 37 children (37%) (26 males and 11 females), and their median age was 4 years; the sepsis group without gastrointestinal involvement included 63 children (32 males and 31 females), and their median age was 3 years; the and control group included 30 children (17 males and 13 females), and their median age was 3.5 years. Regarding baseline demographic data, all groups were age- and sex-matched. DBP was significantly lower in sepsis without gastrointestinal involvement group compared to the control group (*P* = 0.005). Similarly, temperature was significantly higher in the sepsis groups (38.7 ± 0.6 °C and 38.7 ± 0.4 °C for sepsis without and with gastrointestinal involvement, respectively) than in controls (37.1 ± 0.2 °C) (*P* < 0.001). Heart rate and respiratory rate also followed the same trend, with significantly elevated values among sepsis patients compared to controls (*P* < 0.001 for both). However, no significant differences were observed between the groups regarding weight, height, and SBP (Table 1).

**Table 1:** Baseline demographic and clinical characteristics among the studied groups

		Controls (***n*** = 30)	Sepsis group		***P***-value
			Without gastrointestinal involvement (***n*** = 63)	With gastrointestinal involvement (***n*** = 37)	
Age (years)	Median (range)	3.5 (0.33–14)	3 (0.17–14)	4 (0.17–12)	0.061
Gender					
Males	*n* (%)	17 (56.7)	32 (50.8)	26 (70.3)	0.162
Females	*n* (%)	13 (43.3)	31 (49.2)	11 (29.7)	
Weight (kg)	Median (range)	17.5 (5–54)	12 (5–50)	17 (4.5–44)	0.077
Height (cm)	Median (range)	97 (62–156)	85 (58–162)	98 (57–148)	0.054
Weight (SD)					
25th	*n* (%)	5 (16.7)	7 (11.1)	6 (16.2)	0.174
50th	*n* (%)	18 (60)	47 (74.6)	19 (51.4)	
75th	*n* (%)	7 (23.3)	9 (14.3)	12 (32.4)	
Height (SD)					
25th	*n* (%)	1 (3.3)	5 (7.9)	2 (5.4)	0.698
50th	*n* (%)	18 (60)	36 (57.1)	19 (51.4)	
75th	*n* (%)	10 (33.3)	16 (25.4)	10 (27)	
90th	*n* (%)	1 (3.3)	6 (9.5)	6 (16.2)	
SBP	Mean ± SD	96 ± 11	90 ± 16	90 ± 22	0.082
DBP	Mean ± SD	66 ± 8^b^	59 ± 12^a^	59 ± 14	0.005*
Temperature	Mean ± SD	37.1 ± 0.2^b,c^	38.7 ± 0.6^a^	38.7 ± 0.4^a^	< 0.001*
Heart rate	Mean ± SD	97 ± 10^b,c^	142 ± 17^a^	141 ± 21^a^	< 0.001*
Respiratory rate	Mean ± SD	26 ± 6^b,c^	47 ± 13^a^	45 ± 13^a^	< 0.001*

*SBP* systolic blood pressure, *DBP* diastolic blood pressure

^*^Significant *P*-value

^a^Significantly different from the control group

^b^Significantly different from the sepsis with gastrointestinal involvement group

^c^Significantly different from the sepsis without gastrointestinal involvement group

There were no statistically significant differences between septic patients with and without gastrointestinal involvement regarding clinical presentation. Types of complaints (*P* = 0.958) and respiratory support (*P* = 0.371) were all comparable between groups. Gastrointestinal manifestations were present only in the gastrointestinal involvement group (Table 2).

**Table 2:** Cause of admission among septic patients with and without gastrointestinal involvement

		Sepsis group		***P***-value
		Without gastrointestinal involvement (***n*** = 63)	With gastrointestinal involvement (***n*** = 37)	
Complaint				
Pneumonia	*n* (%)	40 (63.5)	26 (70.3)	0.958
GIT (vomiting and diarrhea)	*n* (%)	19 (30.2)	10 (27)	
CNS infection	*n* (%)	3 (4.8)	1 (2.7)	
Polytrauma	*n* (%)	1 (1.6)	0 (0)	
Respiratory support				
None	*n* (%)	35 (55.6)	16 (43.2)	0.371
HFNC	*n* (%)	10 (15.9)	6 (16.2)	
CPAP	*n* (%)	2 (3.2)	0 (0)	
MV	*n* (%)	16 (25.4)	15 (40.5)	
Abdominal distention	*n* (%)	-	30 (81.1)	-
Ileus	*n* (%)	-	11 (29.7)	-
Hematemesis	*n* (%)	-	13 (35.1)	-
Melena	*n* (%)	-	15 (40.5)	-
Bleeding per rectum	*n* (%)	-	14 (37.8)	-

*HFNC* high-flow nasal cannula, *CPAP* continuous positive airway pressure, *MV* mechanical ventilation

**Fig. 1:**
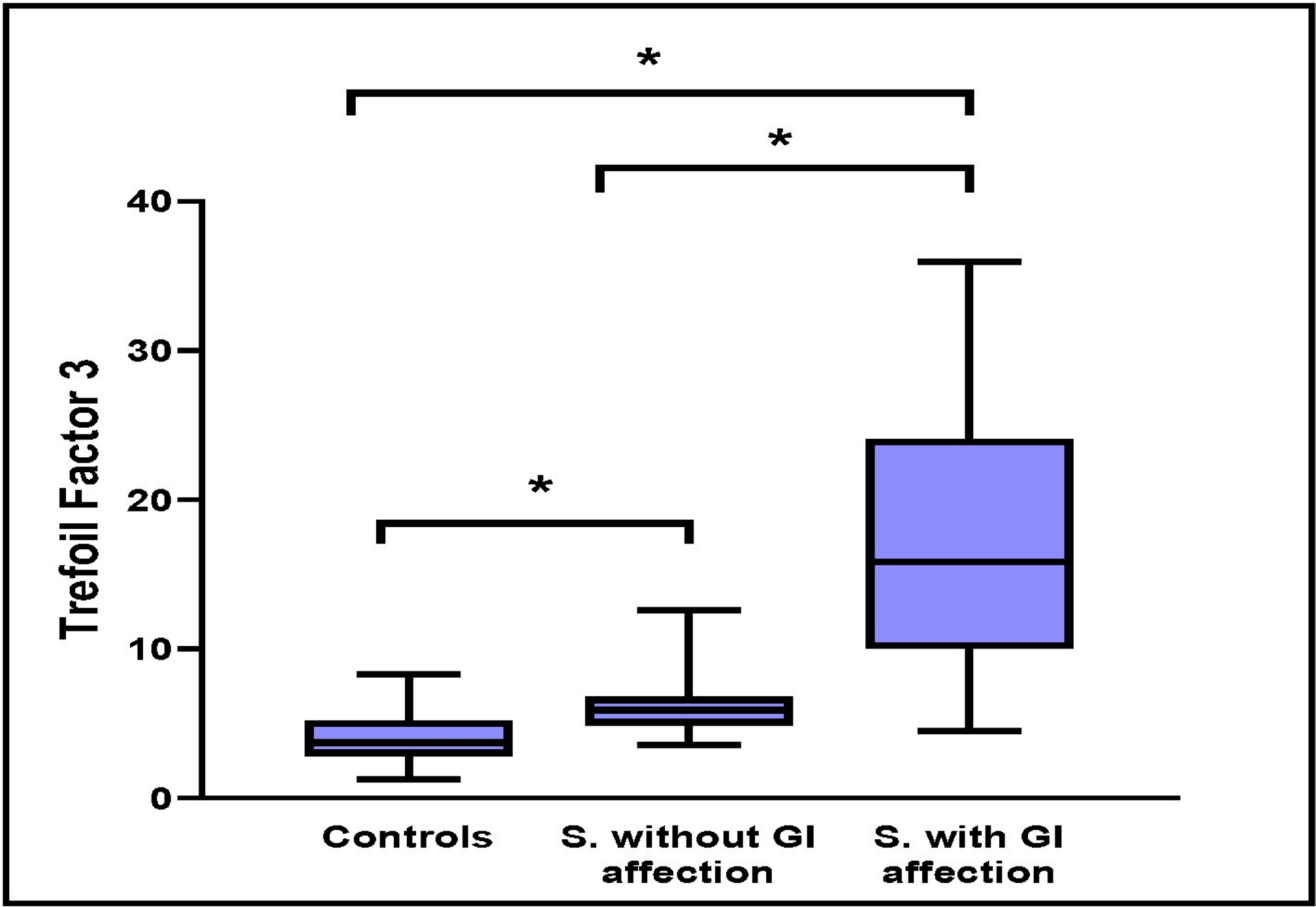
Trefoil factor 3 among the studied groups

Trefoil factor 3 levels were markedly elevated in sepsis with gastrointestinal involvement (median, 15.8; range, 4.49–35.96 ng/mL) compared to sepsis without gastrointestinal involvement (median, 5.89; range, 3.57–12.61 ng/mL) and controls (median, 3.75; range, 1.25–8.29 ng/mL) (*P* < 0.00 l) (Fig. 1).

In septic patients with gastrointestinal involvement, trefoil factor 3 levels were significantly elevated in those with ileus and bleeding per rectum and melena compared to those without. Non-survivors had higher TFF3 levels compared to survivors. Regarding blood culture results, patients with *Klebsiella* had the highest TFF3 levels compared to those with *Staphylococcus aureus* and those with no growth. No significant differences in trefoil factor 3 levels were observed with respect to gender (*P* = 0.832), presenting complaint (*P* = 0.127), type of respiratory support (*P* = 0.055), abdominal distention (*P* = 0.719), or hematemesis (*P* = 0.962) (Table 3).

**Table 3:** Trefoil factor 3 according to other parameters in septic patients with gastrointestinal involvement

	Trefoil factor 3	
	Median (range)	***P***-value
Gender		
Male	15.89 (4.49–35.96)	0.832
Female	15.69 (4.66–32.92)	
Complaint		
Pneumonia	15.64 (4.49–35.96)	0.127
Vomiting and diarrhea	20.47 (6.65–32.92)	
CNS involvement	14.56 (14.56–14.56)	
Respiratory support		
No	11.58 (4.49–24.12)	0.055
HFNC	15.88 (4.66–28.57)	
MV	20.11 (14.36–35.96)	
Abdominal distention		
No	15.83 (6.65–24.12)	0.719
Yes	15.89 (4.49–35.96)	
Ileus		
No	15.08 (4.49–35.96)	0.001*
Yes	24.12 (15.46–32.92)	
Hematemesis		
No	16.34 (4.49–32.92)	0.962
Yes	15.83 (6.65–35.96)	
Melena		
No	15.46 (4.49–19.7)	0.019*
Yes	20.31 (4.86–35.96)	
Bleeding per rectum		
No	14.46 (4.49–19.7)	< 0.001*
Yes	28.42 (15.59–35.96)	
Outcome		
Discharge	15.83 (4.49–28.57)	0.006*
Death	26.66 (15.59–35.96)	
Blood culture		
No growth	15.65 (4.49–24.12)^c^	0.001*
* Staphylococcus aureus*	15.59 (5.28–32.92)^c^	
* Klebsiella*	28.48 (18.7–35.96)^a,b^	

*HFNC* high-flow nasa1 canula, *MV* mechanical ventilation

^*^Significant *P*-value

^a^Significant compared to no growth group

^b^Significant compared to *Staphylococcus aureus* group

^c^Significant compared to *Klebsiella* group

In septic patients without gastrointestinal involvement, trefoil factor 3 showed a significant + ve correlation with duration of hospital stay (*r* = 0.365, *P* = 0.003) and a significant − ve correlation with platelet count (*r* = − 0.336,* P* = 0.007) and ALT levels (*r* = − 0.334, *P* = 0.008). No significant correlations were found with age, SBP, DBP, temperature, heart rate, respiratory rate, PELOD score, TLC, hemoglobin, CRP, urea, creatinine, or AST (All with *P* > 0.05). In septic patients with gastrointestinal involvement, trefoil factor 3 showed a significant positive correlation with PELOD score (*r* = 0.517, *P* = 0.00l) and duration of hospital stay (*r* = 0.333, *P* = 0.046) and significant negative correlations with SBP (*r* = − 0.354, *P* = 0.032), hemoglobin (*r* = –0.451, *P* = 0.005), and platelet count (*r* = − 0.327, *P* = 0.048). No significant correlations were found with age, DBP, temperature, heart rate, respiratory rate, TLC, CRP, urea, creatinine, AST, or ALT (all with *P* > 0.05) (Table 4).

**Table 4:** Correlations between TFF 3 and other parameters in septic patients

	Trefoil factor 3			
	Sepsis without gastrointestinal involvement		Sepsis with gastrointestinal involvement	
	***R***	***P***-value	***r***	***P***-value
Age (years)	− 0.016	0.904	− 0.268	.108
Duration of hospital stay (days)	0.365	0.003*	0.333	0.046*
SBP	− 0.239	0.059	− 0.354	0.032*
DBP	− 0.194	0.128	− 0.282	0.091
Temperature	0.123	0.335	0.243	0.147
Heart rate	0.031	0.808	0.236	0.159
Respiratory rate	0.054	0.676	0.151	0.372
PELOD score	0.164	0.198	0.517	0.001*
TLC	0.158	0.216	0.227	0.178
Hemoglobin	0.026	0.84	− 0.451	0.005*
Platelets	− 0.336	0.007*	− 0.327	0.048*
CRP	0.053	0.678	0.007	0.967
Urea	− 0.163	0.201	0.251	0.134
Creatinine	0.011	0.935	0.196	0.244
AST (U/L)	− 0.191	0.133	− 0.038	0.825
ALT (U/L)	− 0.334	0.008*	0.104	0.539

*SBP* systolic blood pressure, *TLC* total leukocyte count, *DBP* diastolic blood pressure, *r* correlation coefficient, *PELOD* Pediatric Logistic Organ Dysfunction

^*^Significant *P*-value

**Fig. 2:**
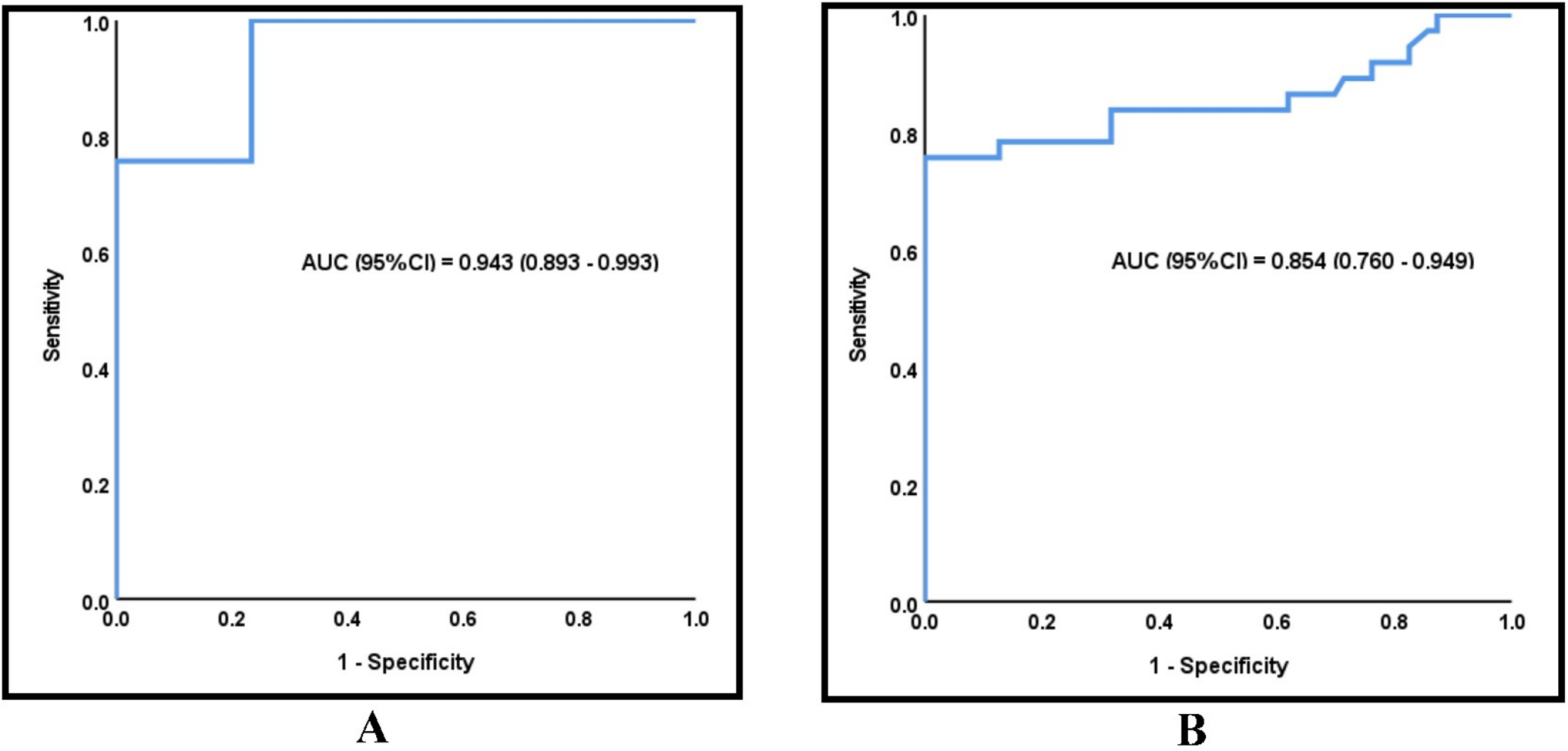
ROC analysis for TFF3 to differentiate **A** between sepsis with gastrointestinal involvement and controls and **B** sepsis with and without gastrointestinal involvement

A ROC curve analysis was done for trefoil factor 3 to differentiate between septic patients with gastrointestinal involvement and controls. It showed a significant AUC of 0.943 with a 95% confidence interval ranging from 0.893 to 0.993, suggesting an excellent ability to differentiate between sepsis with gastrointestinal involvement and controls. The appropriate cutoff was > 4.35 ng/mL, at which sensitivity, specificity, PPV, and NPV were 100%, 76.67%, 84.1%, and 100%, respectively (Fig. 2).

A ROC curve analysis was done for trefoil factor 3 for differentiating between sepsis with and without gastrointestinal involvement. It showed a significant AUC of 0.854 with a 95% confidence interval ranging from 0.760 to 0.949, suggesting a very good ability to differentiate between septic patients with gastrointestinal involvement and septic patients without gastrointestinal involvement. The best cutoff was > 12.61 ng/mL, at which sensitivity, specificity, PPV, and NPV were 75.68%, 100%, 100%, and 87.5%, respectively (Fig. 2).

In the multinomial logistic regression analysis using the control group as the reference category, trefoil factor 3 (TFF3) emerged as an important predictor of sepsis outcomes, controlling for age and gender. For patients with sepsis without gastrointestinal (GI) involvement, higher TFF3 levels were significantly relates to high odds of sepsis (OR = l.814, 95% CI 1.282–2.567, *P* = 0.001). In sepsis with gastrointestinal involvement group, TFF3 showed an even greater association (OR = 3.095, 95% CI 2.044–4.686, *P* < 0.001) (Table 5).

**Table 5:** Multinomial logistic regression analysis to predict sepsis

Outcome	OR (95% CI)	*P*-value
**Sepsis without gastrointestinal involvement**		
Age (years)	0.907 (0.789–1.044)	0.173
Gender	0.528 (0.177–1.576)	0.252
Trefoil factor 3	1.814 (1.282–2.567)	0.001*
**Sepsis with gastrointestinal involvement**		
Age (years)	1.213 (0.989–1.486)	0.063
Gender	0.077 (0.011–0.556)	0.011
Trefoil Factor 3	3.095 (2.044–4.686)	< 0.001*

*OR* odds ratio, *95% CI* confidence interval 95%

^*^Significant *P*-value

A multivariate linear regression analysis was performed to identify independent predictors of the PELOD score. Trefoil factor 3 emerged as the strongest independent factor, with each unit increase in its level associated with a 0.281-point increase in the PELOD score (*B* = 0.281, 95% CI 0.174 to 0.389, *P* < 0.001), controlling for age and gender (Table 6).

**Table 6:** Multivariate linear regression analysis to predict PELOD score

	***B* (95% CI)**	***P*-value**
Age (years)	− 0.248 (− 0.501–0.005)	0.054
Gender	0.354 (− 1.417–2.125)	0.693
Trefoil factor 3	0.281 (0.174–0.389)	< 0.001*

^*^Significant *P*-value < 0.05

*CI* confidence interval, *B* regression coefficient

## Discussion

Trefoil factor 3 (TFF3) is a small peptide that is essential for gastrointestinal protection and mucosal repair, while also maintaining the integrity of intestinal barrier [10].

In the current study, trefoil factor 3 levels were markedly elevated in sepsis with gastrointestinal involvement than sepsis without gastrointestinal involvement and controls.

This is consistent with Ma et al. [11], who reported that serum TFF3 levels measured prior to the development of gastrointestinal failure were significantly higher in group C (sepsis with GIF) compared to group A (controls) and group B (sepsis without GIF). Furthermore, no statistically significant difference was observed between groups A and B.

In the study conducted by Žurek et al. [3] which evaluated TFF3 as a biomarker of intestinal epithelial injury during sepsis, the authors suggested that TFF3 levels were unable to distinguish between different septic states until significant organ dysfunction became evident. This finding was reinforced by the significantly elevated and distinctive TFF3 levels detected in patients with multiple organ dysfunction syndrome and higher PELOD scores. These results suggest that gastrointestinal injury is partially present in sepsis associated with multiple organ dysfunction. Furthermore, gastrointestinal involvement may develop or worsen as sepsis advances.

Notably, Ng et al. [12] investigated TFF3 in preterm infants as a biomarker for distinguishing NEC from septicemic and control cases. Plasma levels of the gut barrier marker TFF3 were significantly higher in infants with NEC compared with those with septicemia or healthy controls. Importantly, TFF3 showed no correlation with nonspecific systemic inflammatory markers, highlighting its specific association with intestinal injury rather than with the overall systemic inflammatory response. Moreover, TFF3 has been proposed as a potential therapeutic option for NEC, as studies in experimental rat models demonstrated that TFF3 administration mitigated intestinal tissue injury [6].

During sepsis, these physiological mechanisms become impaired, thereby contributing to the development of organ failure. Increased plasma TFF3 concentrations are regarded as indicators of intestinal epithelial injury and have been reported in children with necrotizing enterocolitis, as well as serving as markers of disease severity in colitis. Although gut barrier dysfunction has long been implicated in the pathophysiology of sepsis, the temporal behavior of plasma TFF3 levels has not been fully explored in pediatric patients with sepsis [13].

In the current study, in septic patients without gastrointestinal involvement, trefoil factor 3 demonstrated a significant inverse correlation with platelet count and alanine aminotransferase levels and a significant positive relationship with length of hospital stay. In contrast, in septic patients with gastrointestinal involvement, trefoil factor 3 showed significant positive associations with PELOD score and period of hospitalization, along with significant negative correlations with systolic blood pressure, hemoglobin concentration, and platelet count.

This was partially agreed with Žurek et al. [3], who found that patients with multiple organ dysfunction syndrome, higher PELOD scores, or prolonged hospitalization exhibited significantly elevated TFF3 levels. However, no differences in TFF3 level dynamics were detected between survivors and non-survivors. This result may have been influenced by early dropout among non-surviving patients.

Our findings were also consistent with Sarangam et al. [14], who studied intestinal injury biomarkers as predictors of mortality in pediatric severe malaria. In their study, TFF3 was a significant predictor in-hospital mortality (OR, 4.4; 95% CI, 2.7–7.3) and was also related to post-discharge mortality (OR, 2.43; 95% CI, 1.1–4.8).

Similarly, in adult study, by Sun et al. [15], TFF3 level was significantly elevated in patients who had sepsis at ICU admission and after 3 days of treatment compared with healthy controls. Among septic patients, those with septic shock exhibited significantly greater TFF3 concentrations than patients with uncomplicated sepsis. Serum TFF3 levels showed a positive correlation with sepsis severity, including multi-organ failure scores and inflammatory mediators. Across all septic patients, TFF3 concentrations increased from admission to the third day of ICU care, and elevated levels on day three were strongly associated with poor clinical outcomes.

In the current study, ROC curve analysis was done for trefoil factor 3 to differentiate between septic patients with gastrointestinal involvement and controls.

Our findings are similar to the findings of Žurek et al. [3], who established that TFF3 effectively differentiated patients from controls, as well as those with multiple organ dysfunction syndrome or higher PELOD scores. The optimal cutoff values yielding the highest discriminative performance ranged from 1.2 to 2.5 nmol/mL.

In the current study, multivariate linear regression analyses were performed to identify independent predictors of the PELOD score. TFF3 emerged as the most significant independent predictor, with each unit increase in its level corresponding to a 0.281-point rise in the PELOD score (*B* = 0.281, 95% CI 0.174–0.389, *P* < 0.001), after adjusting for age and gender.

This is consistent with Ma et al. [11], who reported a significant association between serum TFF3 concentrations and GIT function scores (*r* = 0.712). Additionally, Cox proportional hazards analysis revealed that serum TFF3 levels measured at the onset of GI failure and 48 h later acted as prognostic markers in critical ill pediatric patients (hazard ratios of l.443 and 1.872, respectively).

Finally, these findings demonstrate that increased serum TFF3 levels are linked to sepsis severity, GIT involvement, multiple organ dysfunction, and clinical outcomes in septic patients. Consequently, TFF3 could function as a valuable indicator of gastrointestinal injury in pediatric sepsis and may contribute to mucosal protection and tissue repair.

## Limitations

This study has several limitations. First, it was conducted at a single center, which may limit the generalizability of the findings to other populations and clinical settings. Second, the sample size was relatively small, and patients with different etiologies were included, which may introduce potential selection bias. Third, TFF3 levels were measured at a single time point upon admission, and serial measurements were not performed; therefore, the temporal dynamics of TFF3 in relation to disease progression could not be assessed. Additionally, the potential impact of nutritional status and feeding practices in the PICU on TFF3 levels was not evaluated.

## Conclusion

TFF3 is a valuable tool in the PICU setting, providing an objective measure of intestinal damage and helping clinicians anticipate, diagnose, and manage gastrointestinal failure in vulnerable pediatric patients.

## Data Availability

Data are available on reasonable request from corresponding author

## Abbreviations

MODS: Multiple organ dysfunction syndrome
OD: Optical density
PICUs: Pediatric Intensive Care Units
PELOD: Pediatric Logistic Organ Dysfunction
TFF3: Trefoil factor 3
